# Supplementary material for: Mechanical mismatch-driven rippling in carbon-coated silicon sheets for stress-resilient battery anodes
Source: Nat Commun. 2018 Jul 26;9:2924. doi: 10.1038/s41467-018-05398-9 (PMC6062545; doi:10.1038/s41467-018-05398-9)
Supplement: Supplementary file 1 — Supplementary Information [file 41467_2018_5398_MOESM1_ESM.pdf]

Supplementary Information

Mechanical mismatch-driven rippling in carbon-coated silicon sheets for stress-resilient battery anodes

Ryu et al.

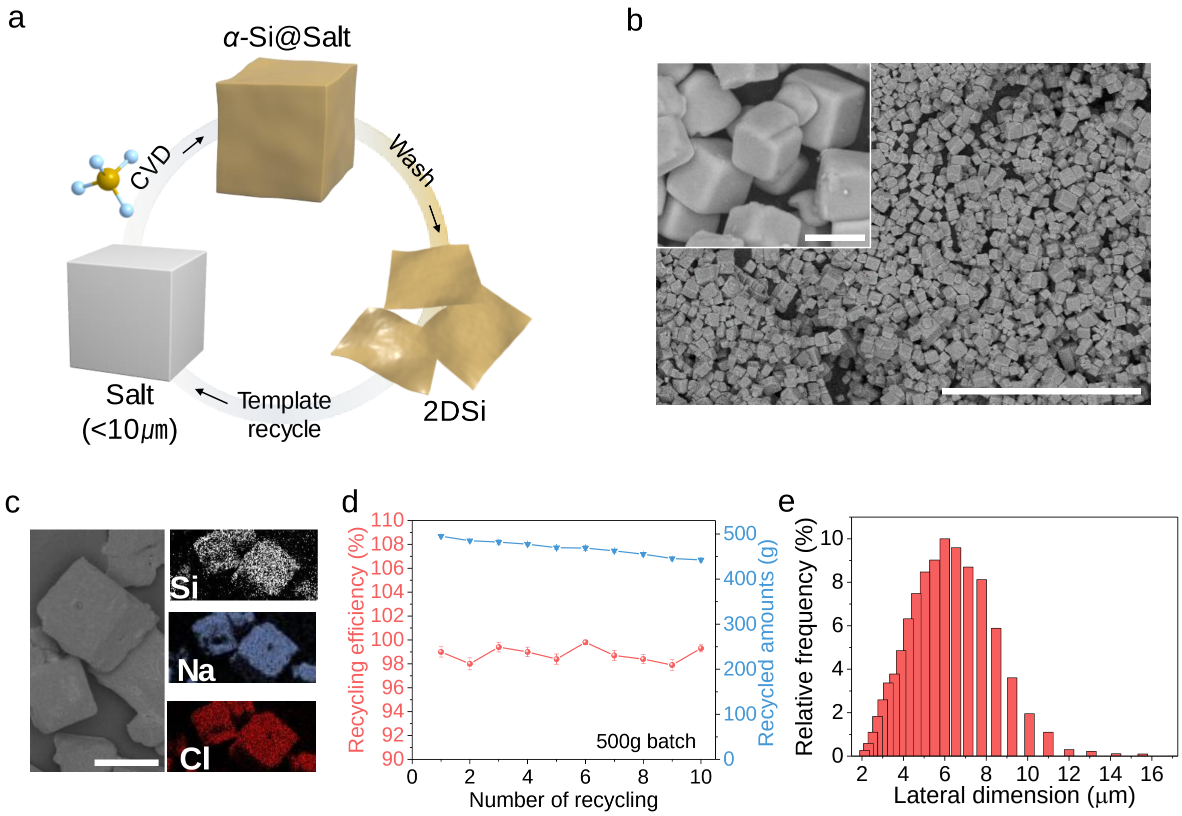

Supplementary Figure 1. Synthesis of 2DSi anode via recyclable salt templating method. (a) Schematic illustration showing synthetic process of 2DSi. (b) SEM images of 10 μm-sized cubic crystal of NaCl. (c) SEM image and the corresponding EDS image of Si@NaCl. (d) Recycling efficiency for 10 cycles and the amount of salt recycled starting from initial amount of 500 g. (e) Particle size distribution of 2DSi materials based on the SEM images. The scale bars are 100 μm (inset: 5 μm) and 5 μm for (b) and (c), respectively.

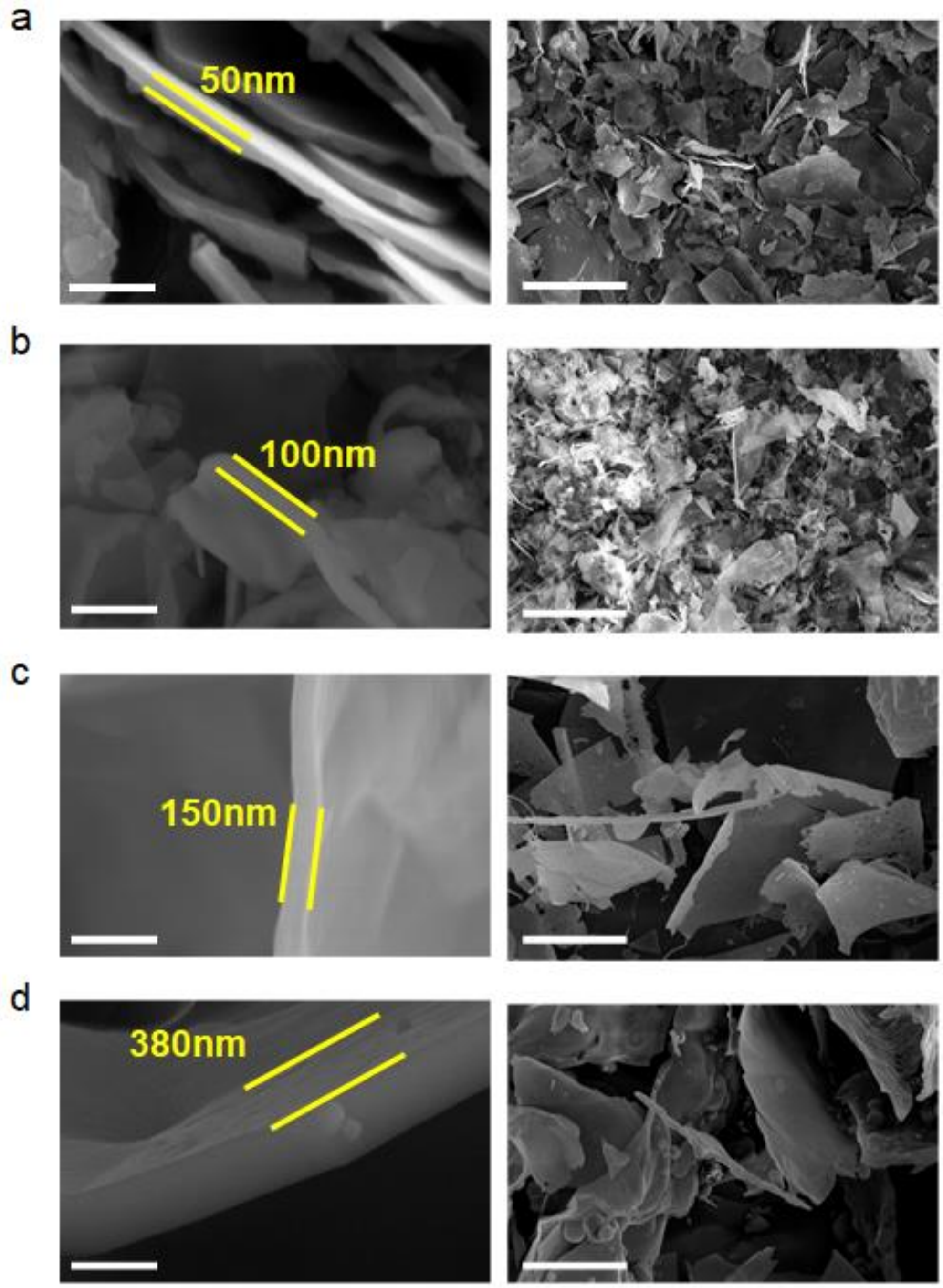

Supplementary Figure 2. Variable Thickness of 2DSi. SEM images of (a) 50 nm-thick, (b) 100 nm-thick, (c) 150 nm-thick, and (d) 380 nm-thick 2DSi nanosheets. The scale bars are 500 nm for left column and 10 μm for right column.

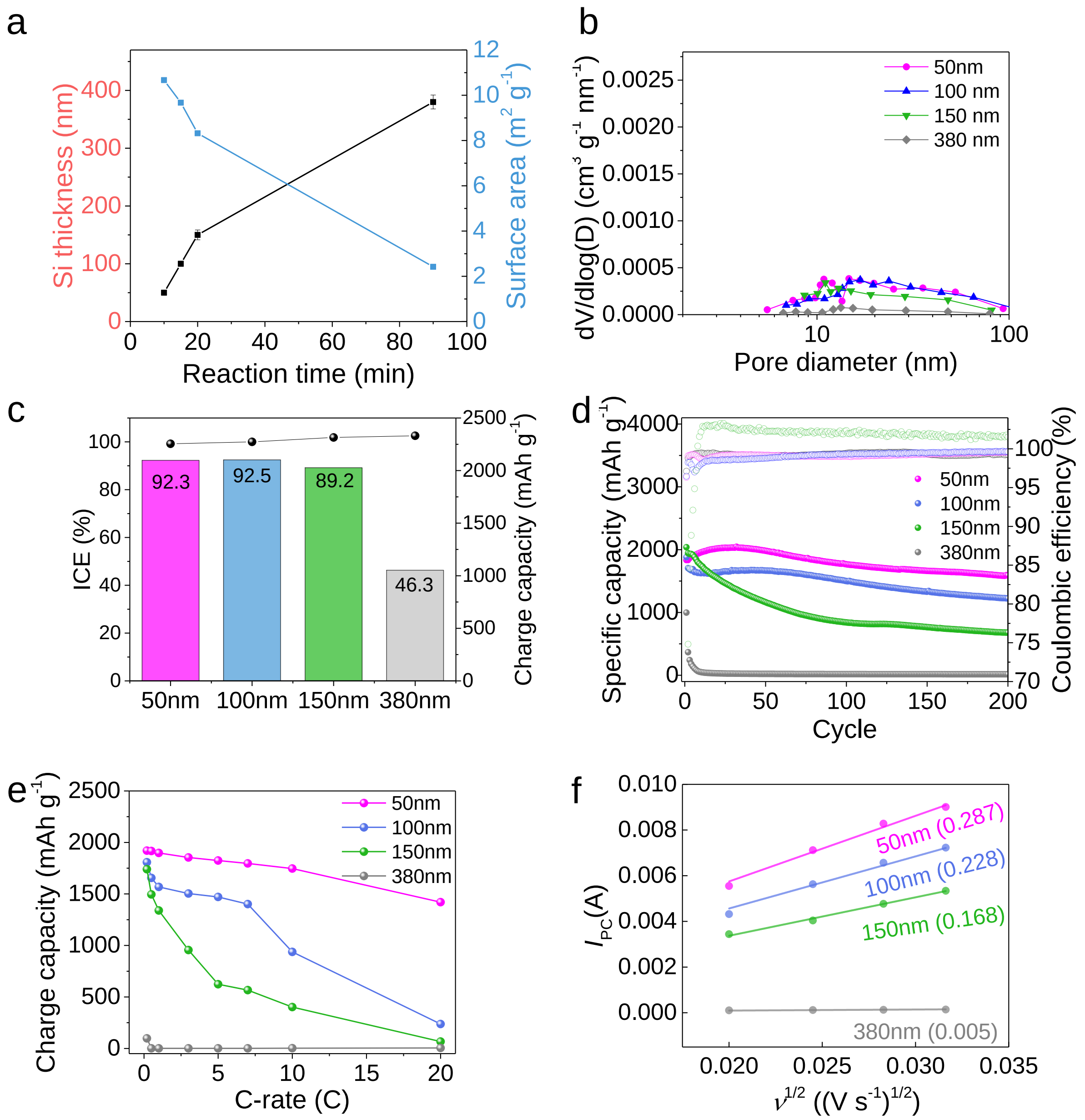

Supplementary Figure 3. Characterization of various 2DSi@C electrodes. (a) CVD reaction time-dependent thickness of 2DSi and their surface areas. (b) BJH pore size distribution curves for 2DSi with different thickness. Thickness-dependent properties: (c) Charge capacities and ICE values, (d) cycle retention at 0.2 C-rate with Coulombic efficiency, (e) charge capacities at different C-rate, and (f) electroactive-surface-area calculation plots based on CV results.

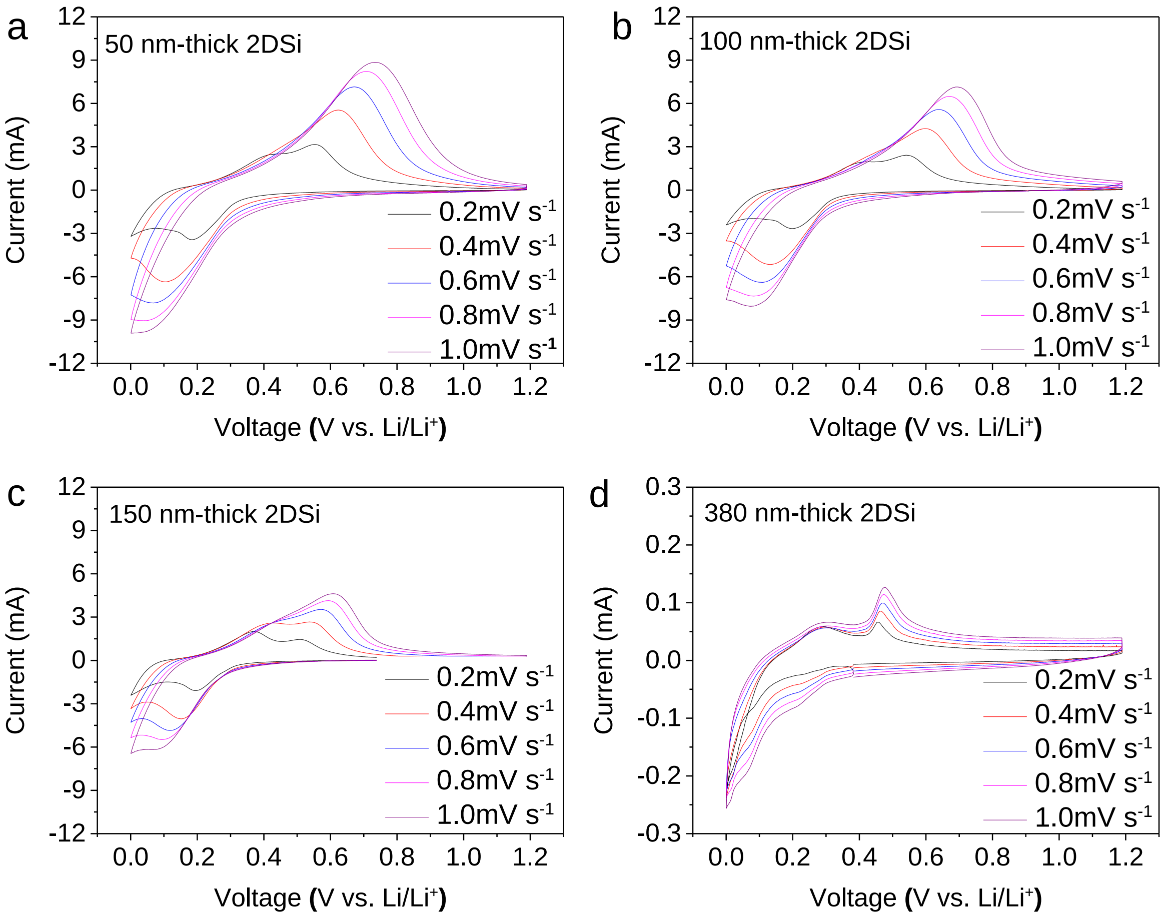

Supplementary Figure 4. CV results of 2DSi electrodes with different thickness. CV curves with a variation of scan rate from 0.2 to 1.0 mV s^{-1} of (a) 50 nm, (b) 100 nm, (c) 150 nm, and (d) 380 nm-thick 2DSi@C electrodes.

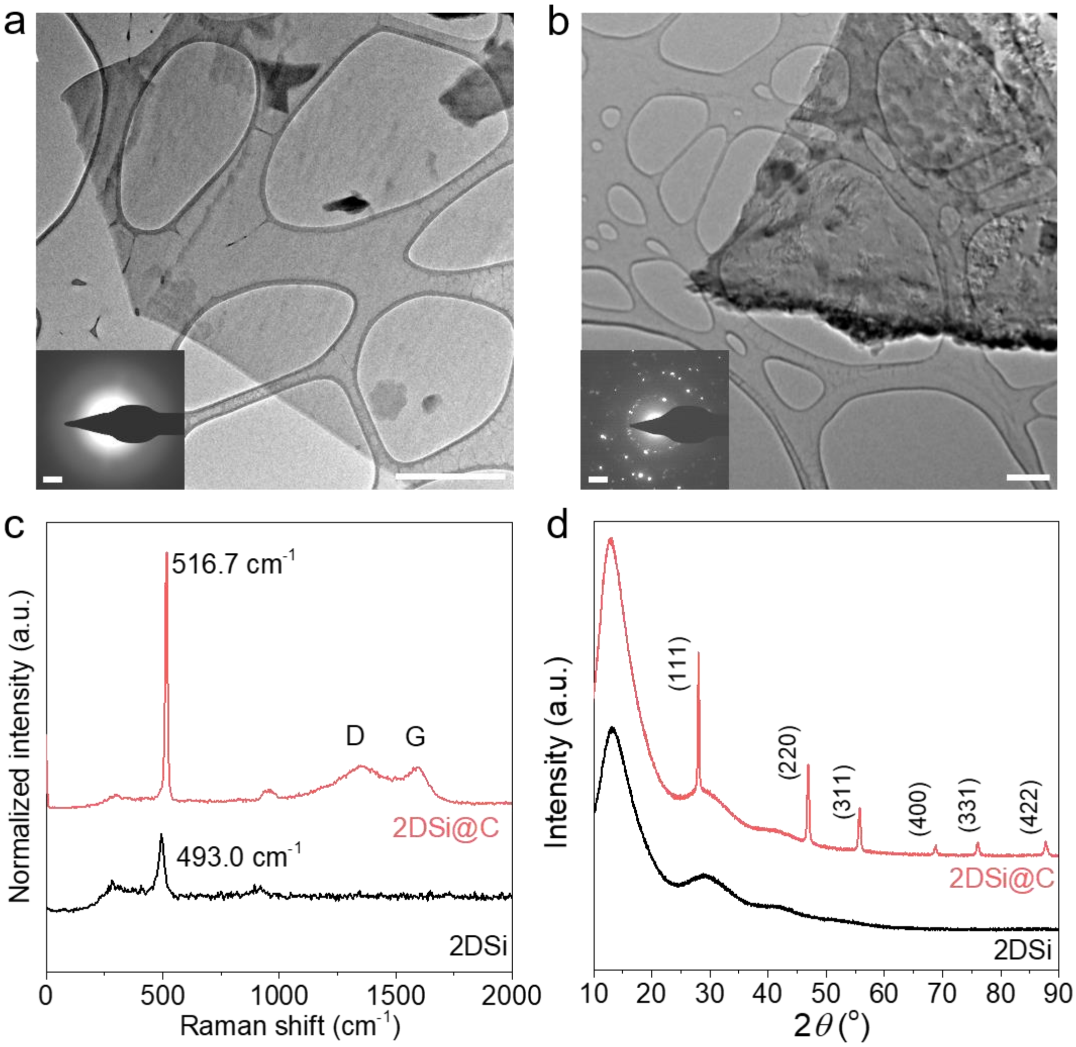

Supplementary Figure 5. Basic information on 2DSi and 2DSi@C. The TEM images of (a) 2DSi and (b) 2DSi@C (Inset. Corresponding SAED). (c) Raman spectrum and (d) XRD pattern of 2DSi (black) and 2DSi@C (red), which shows phase transition of amorphous Si to polycrystalline Si after carbon coating due to high temperature annealing at 900℃. The scale bars are 500 nm for TEM image and 2/nm for SAED.

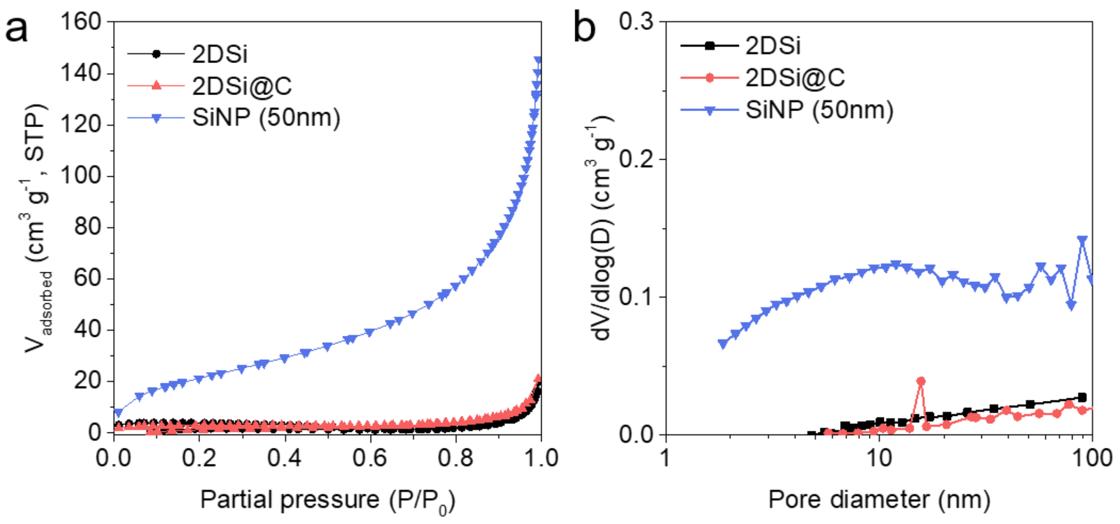

Supplementary Figure 6. Nitrogen sorption analysis. (a) Nitrogen adsorption-desorption isotherms and (b) BJH pore size distribution curves of 2DSi (black), 2DSi@C (red), and Si nanoparticle (SiNP with a size of 50nm, blue), which show non-porous structure of as-prepared 2DSi before/after carbon coating compared to commercial SiNP with a same size of 50 nm.

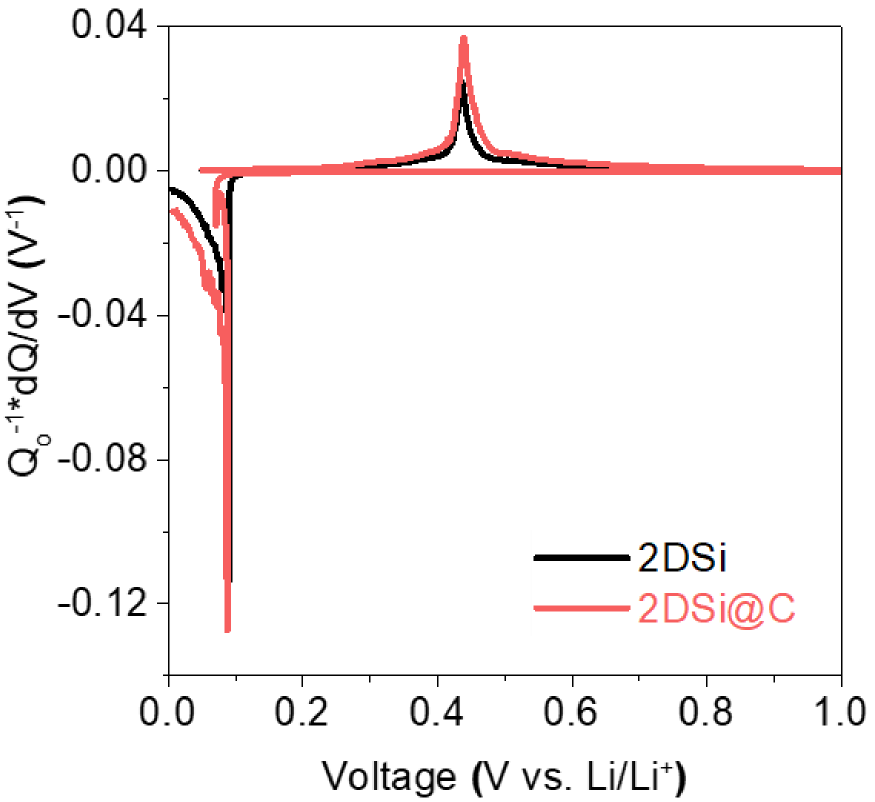

Supplementary Figure 7. Identification of redox potentials. Differential capacity curves of 2DSi (black) and 2DSi@C (red). Even after carbon coating, redox potentials for Li-Si alloying and dealloying process have not changed, indicating that different types of Li storage behaviors originated from carbon coating do not occur.

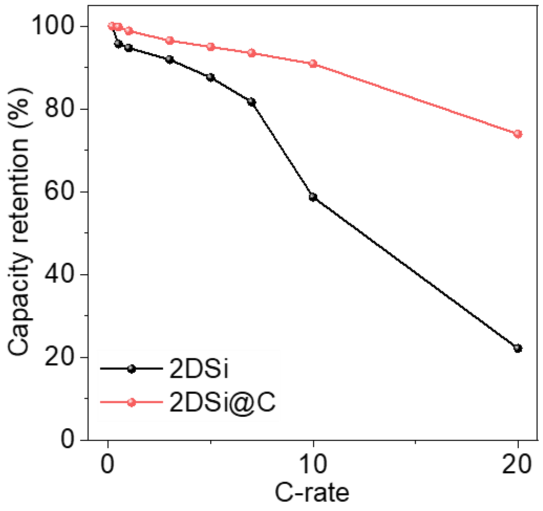

Supplementary Figure 8. Capacity retention plot at various C-rate results of 2DSi-based electrodes. Capacity retentions of 2DSi (black) and 2DSi@C (red) at 0.2-20 C-rate for both discharge/charge.

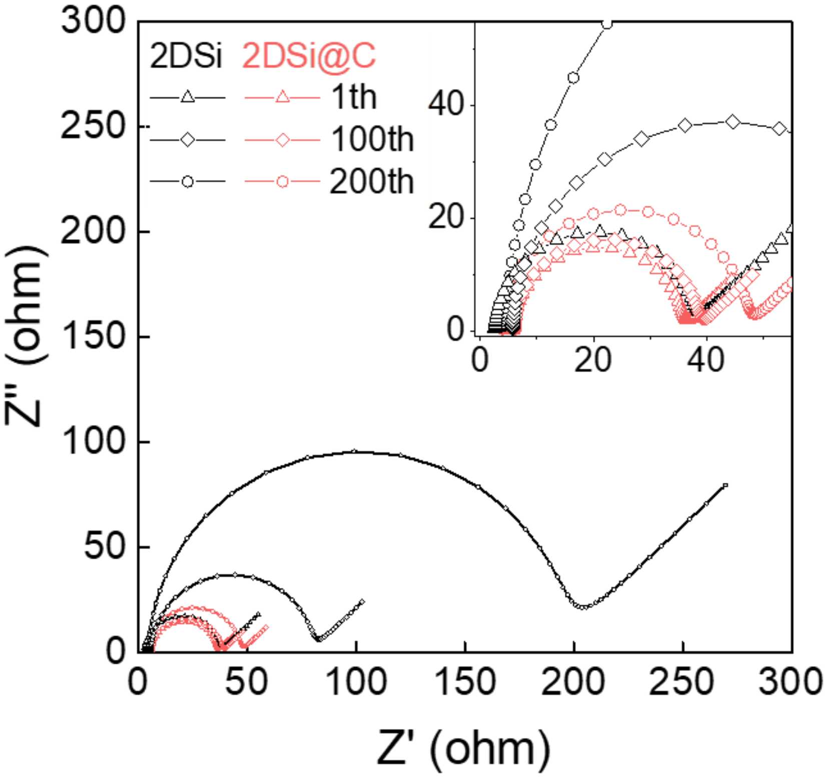

Supplementary Figure 9. ***Ex situ*** **EIS results.** Impedance spectra of 2DSi and 2DSi@C half-cell after 1st, 100th cycles and 200th cycles.

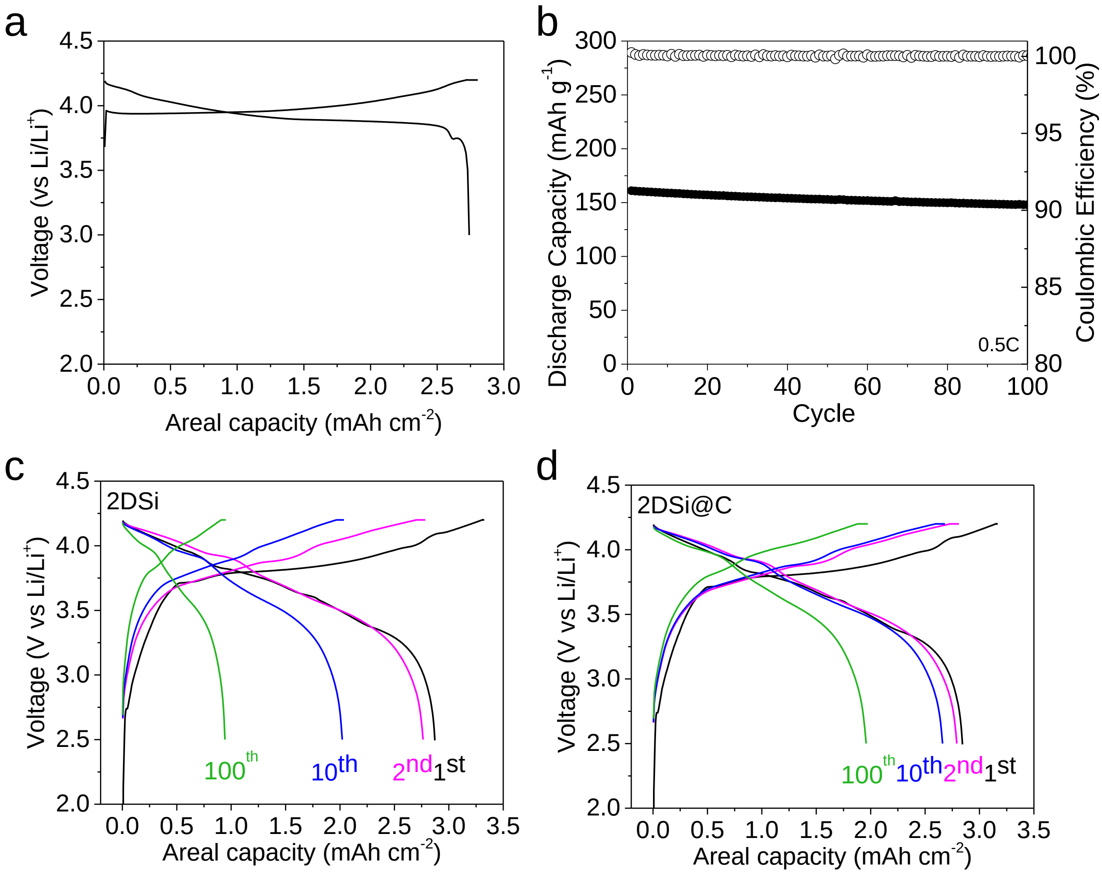

Supplementary Figure 10. Cathode and full cell evaluation. Voltage profiles at initial cycle at 0.1 C-rate (a) and cycle retention at 0.5 C-rate for $LiCoO_2$ cathode (b) in the potential window of 3.0-4.3V. Voltage profiles of $LiCoO_2$//2DSi (c) and $LiCoO_2$//2DSi@C (d) full cells during cycles in the potential window of 2.5-4.2V.

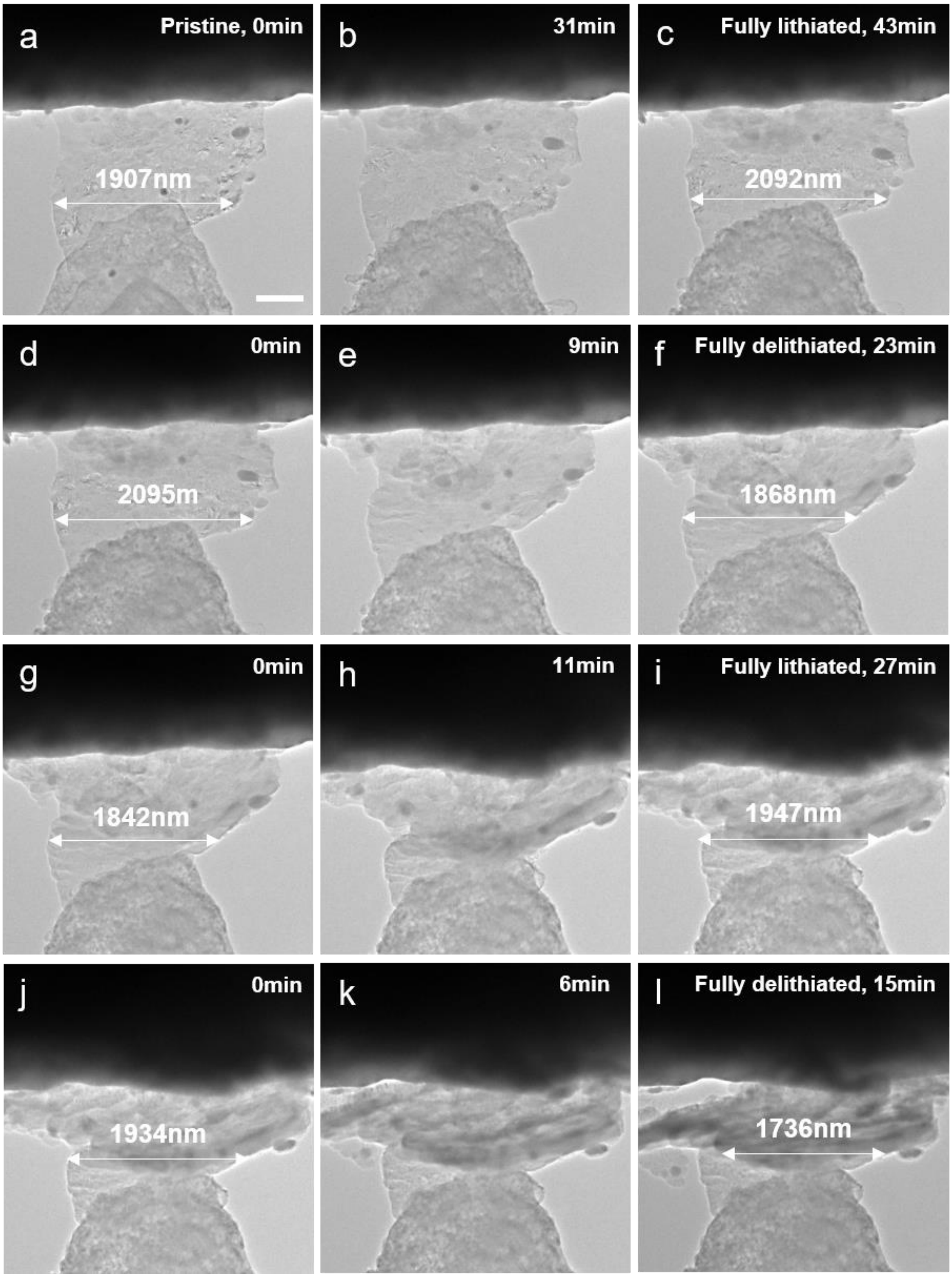

Supplementary Figure 11. Time-resolved TEM images during multiple lithiation /delithiation process of a single 2DSi@C nanosheet. (a-c) First lithiation, (d-f) First delithiation, (g-i) second lithiation, and (j-l) second delithiation. Scale bar is 500 nm.

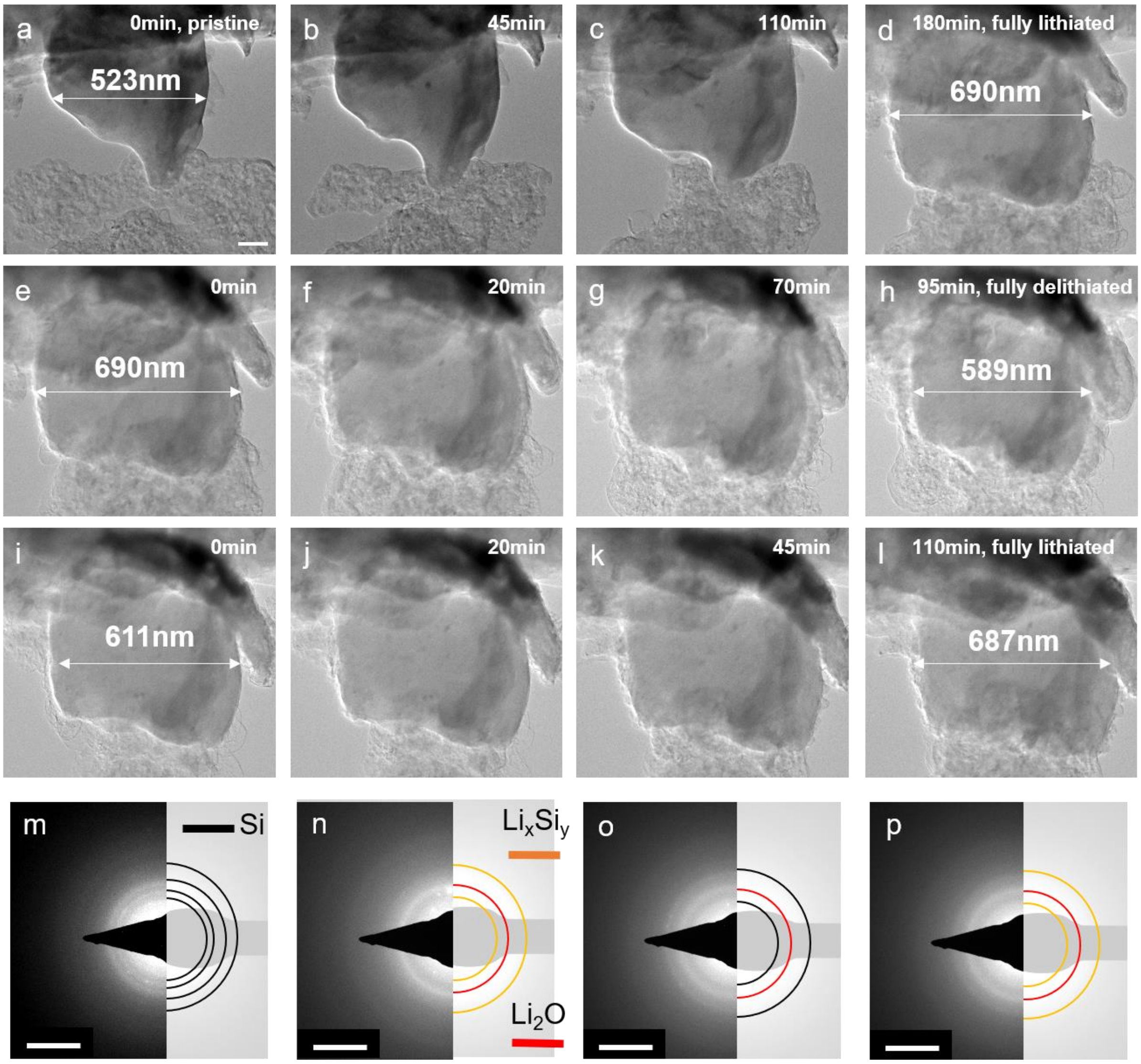

Supplementary Figure 12. Time-resolved TEM images during multiple lithiation/delithiation process of a single 2DSi nanosheet. (a-d) First lithiation, (e-h) First delithiation, and (i-l) second lithiation. SAED patterns of (m-p) are corresponding to the states of (m) Pristine, (n) full lithiation, (o) full delithiation, and (p) full lithiation, respectively. The scale bars are 100 nm for TEM images and 5/nm for SAED patterns, respectively.

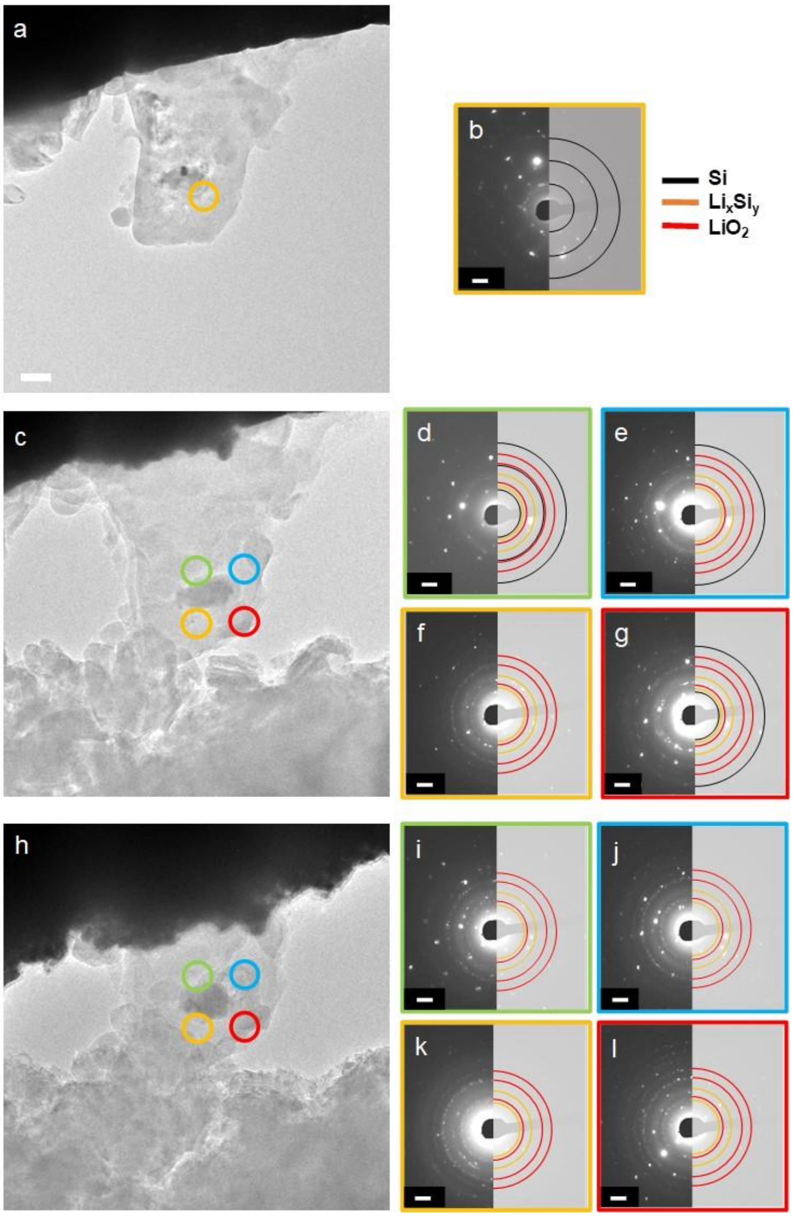

Supplementary Figure 13. Li-ion diffusion path of 2DSi@C. Li-ion pathway during lithiation is investigated by comparing the SAED patterns in a single 2DSi@C nanosheet. (a, c, h) Featured TEM images during lithiation. Each image shows pristine, mid-lithiated, and fully lithiated state, respectively. Each colored circle is corresponding to SAED patterns consistent with colored frames. The SAED patterns of (b), (d-g) and (i-l) correspond to the featured TEM images of (a), (c), and (h), respectively. Li-ion path is similar whether carbon was coated or not, but quite faster due to the carbon layers, as we expected. The scale bars are 100 nm for TEM images and 2/nm for SAED patterns, respectively.

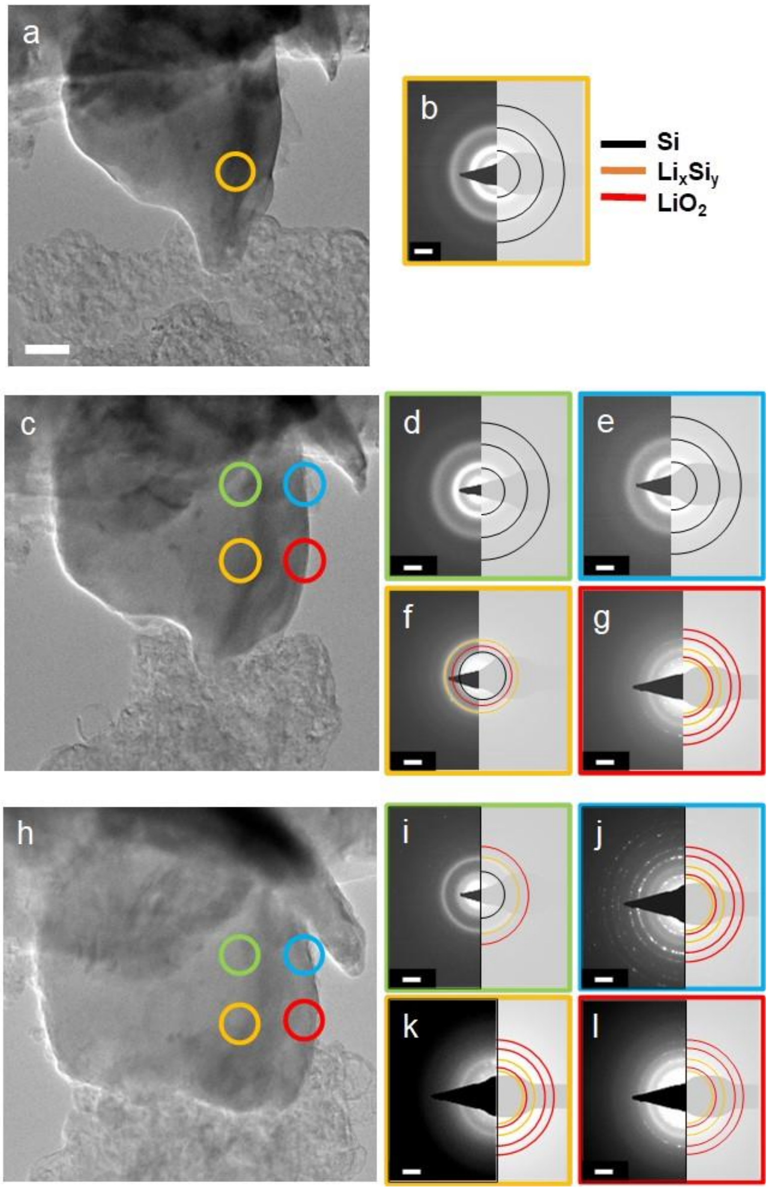

Supplementary Figure 14. Li-ion diffusion path of 2DSi. Li-ion pathway during lithiation is investigated by comparing the SAED patterns in a single 2DSi nanosheet. (a, c, h) Featured TEM images during lithiation. Each image shows pristine, mid-lithiated, and fully lithiated state, respectively. Each colored circle is corresponding to SAED patterns consistent with colored frames. The SAED patterns of (b), (d-g) and (i-l) correspond to the featured TEM images of (a), (c), and (h), respectively. As ring patterns for Li_xSi_y are obviously distinguishable in individual SAED patterns, Si in the position marked by green circle has remained unreacted while other sites undergo Li-Si alloying reaction. Therefore, Li-ion diffusion in 2DSi prefer to propagate along the edges rather than crossing the particle. The scale bars are 100 nm for TEM images and 2/nm for SAED patterns, respectively.

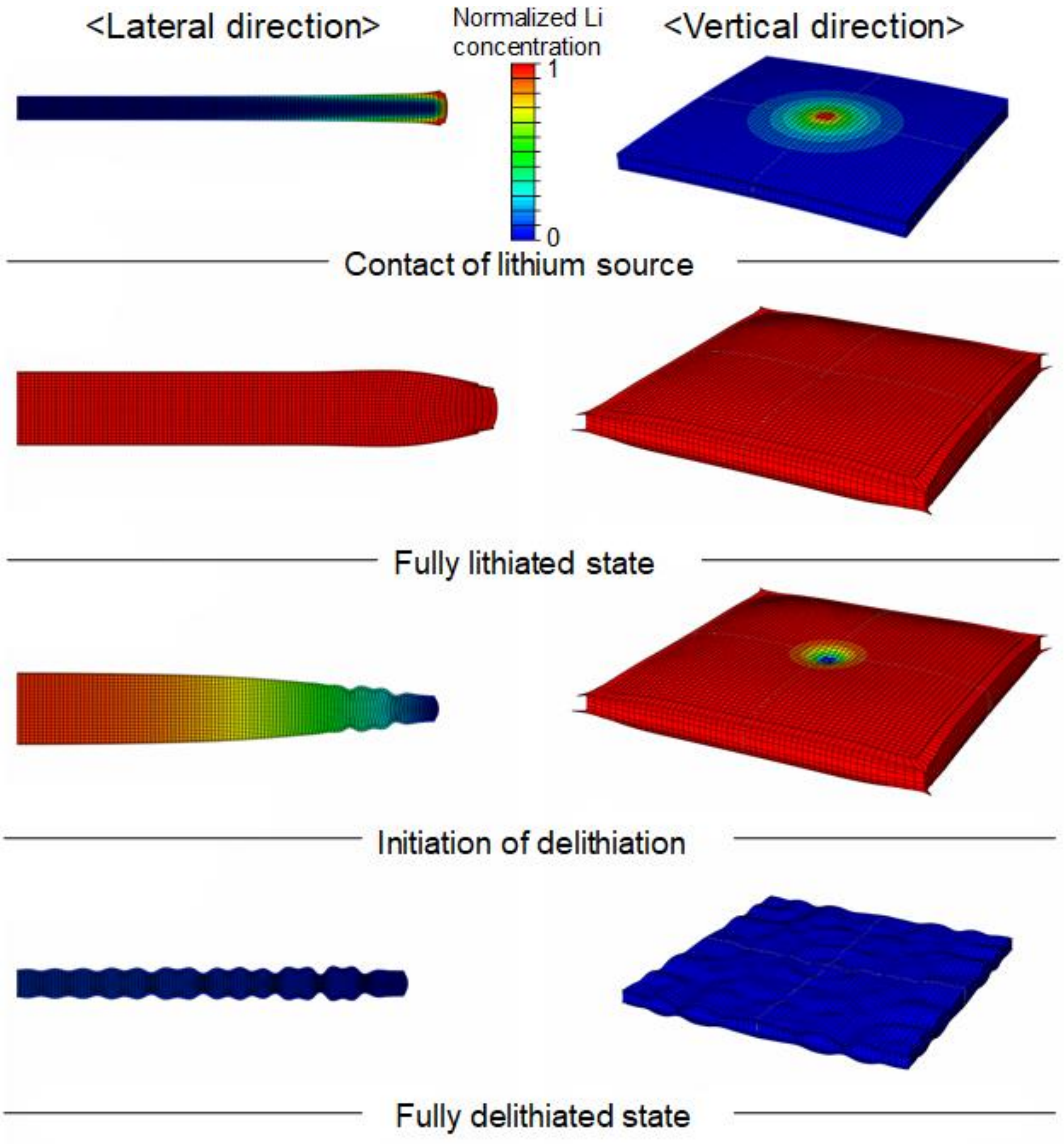

Supplementary Figure 15. Structural modeling upon different positions of lithium source. As the lithium source approaches the 2DSi@C in lateral and vertical directions, both set-ups result in ripple formation over the sheet.

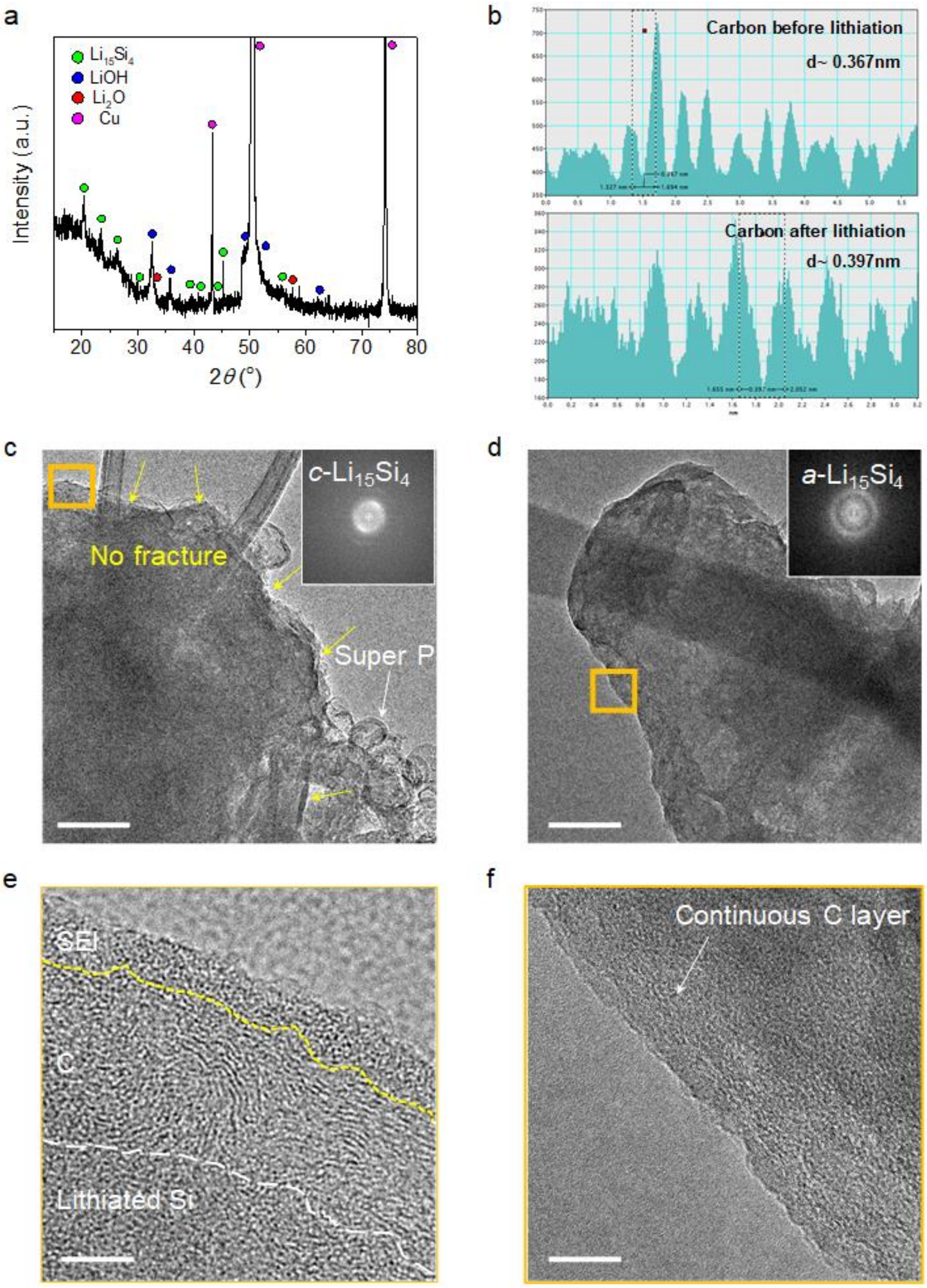

Supplementary Figure 16. ***Ex situ*** **TEM analysis on fully lithiated 2DSi@C.** (a) XRD pattern of fully lithiated 2DSi@C electrode. (b) Comparison of carbon interspacing values before and after lithiation. (c-f) TEM images of fully lithiated 2DSi@C sheet, which demonstrates no obvious fracture or break was not found on the carbon layers and, rather dense and uniform SEI layers were formed. The scale bars are 200 nm, 200 nm, 5 nm, and 10 nm for (c-f), respectively.

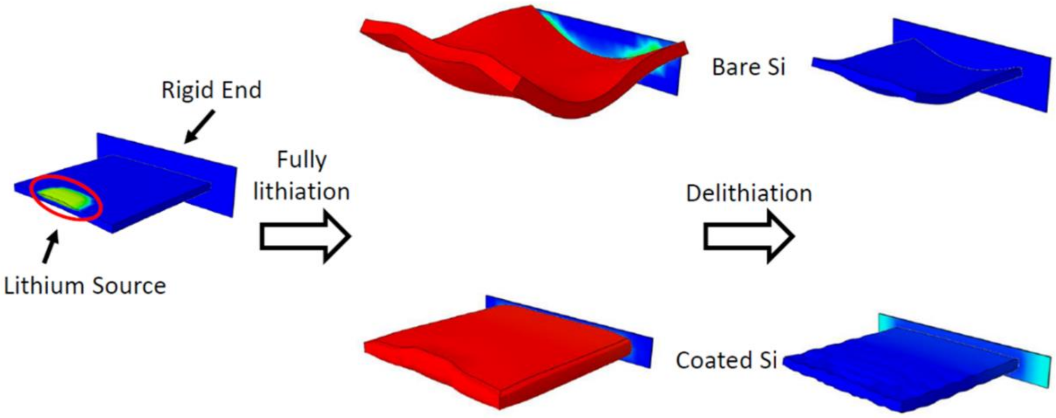

Supplementary Figure 17. Chemo-mechanical modeling of 2DSi and 2DSi@C. The rippling phenomenon can only be found in 2DSi@C during delithiation step.

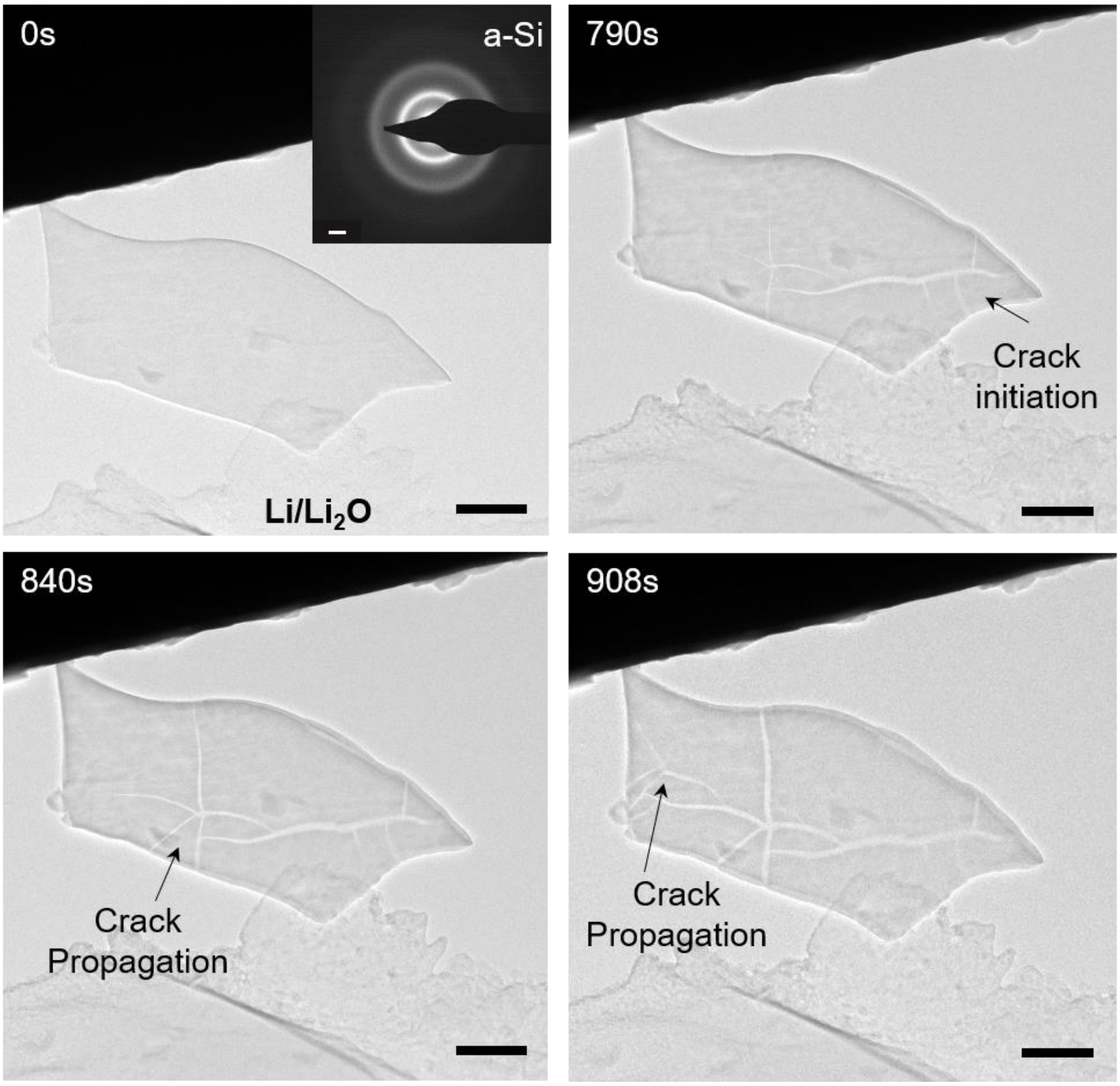

Supplementary Figure 18. *In situ* TEM observations on 2DSi with larger size during lithiation. 2DSi has an amorphous nature before lithiation. During lithiation, cracks formed near at the Li/Li_2O end and it propagated to the counter W electrode. The scale bars are 500 nm for TEM images and 2/nm for SAED patterns, respectively.

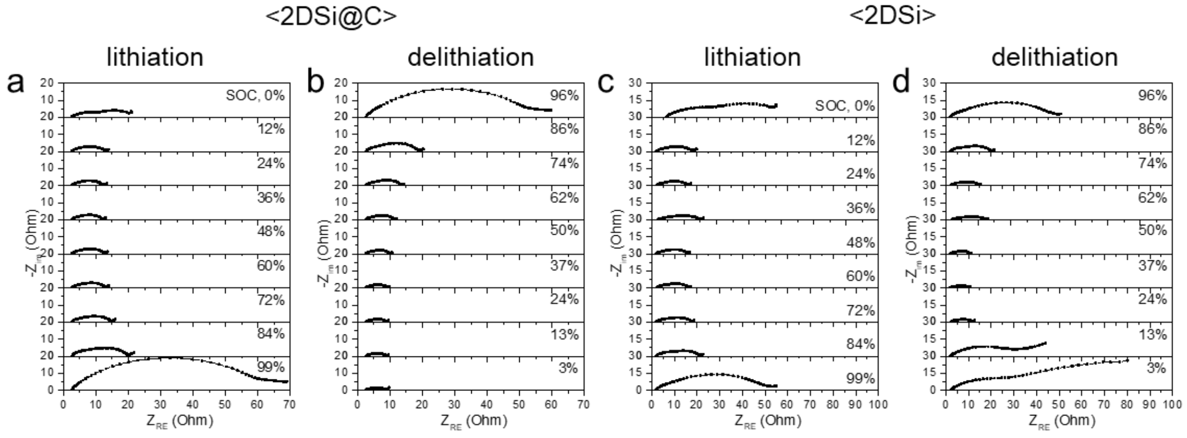

Supplementary Figure 19. Comparison of Impedance between 2DSi and 2DSi@C during 1st cycle. Impedance spectra measured at each SOC during (a and c) lithiation and (b and d) delithiation of 2DSi and 2DSi@C, respectively.

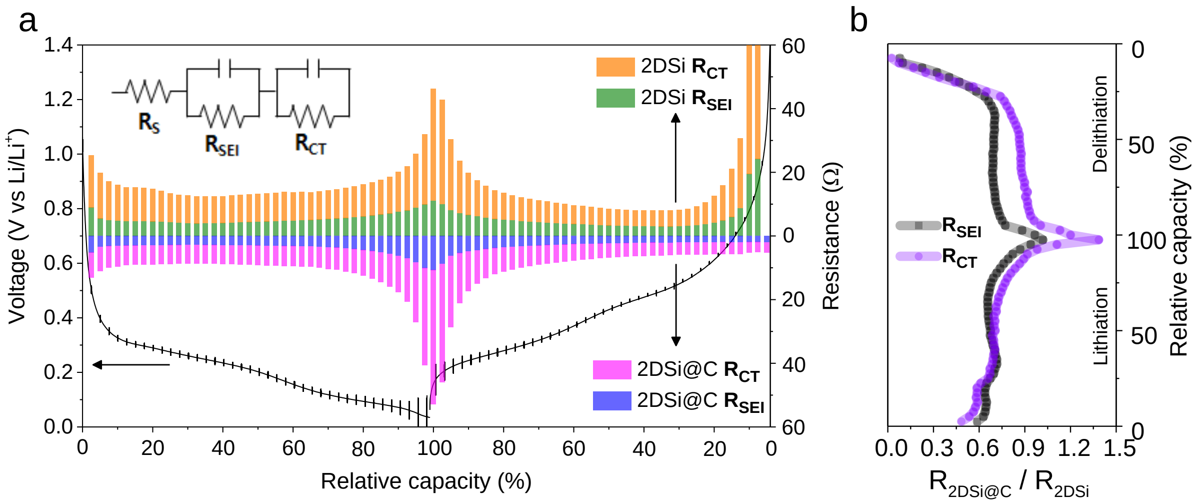

Supplementary Figure 20. Comparison of fitted values for charge transfer and SEI resistances between 2DSi and 2DSi@C during 1st cycle. (a) Comparison of polarization resistance (R_{ct} and R_{SEI}) changes during 1st cycle at a rate of 0.1 C. (b) The resistance ratio ($R_{2DSi@C}/R_{2DSi}$) versus SOC plot.

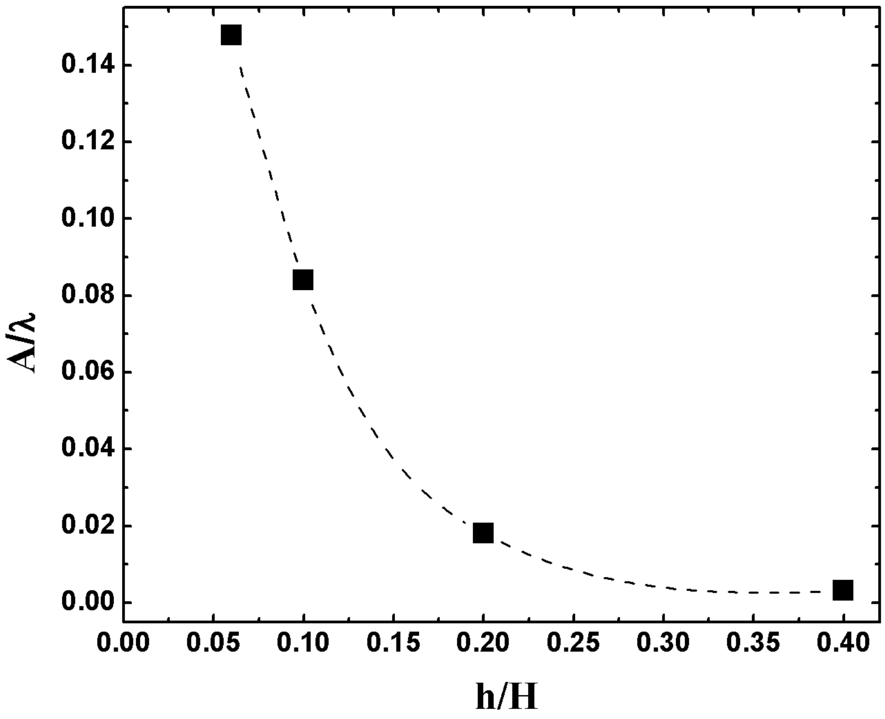

Supplementary Figure 21. Coating thickness dependent rippling structure analysis. The rippling amplitude as a function of the coating thickness, obtained by direct numerical simulations. Here H is the thickness of the Si nanosheet.

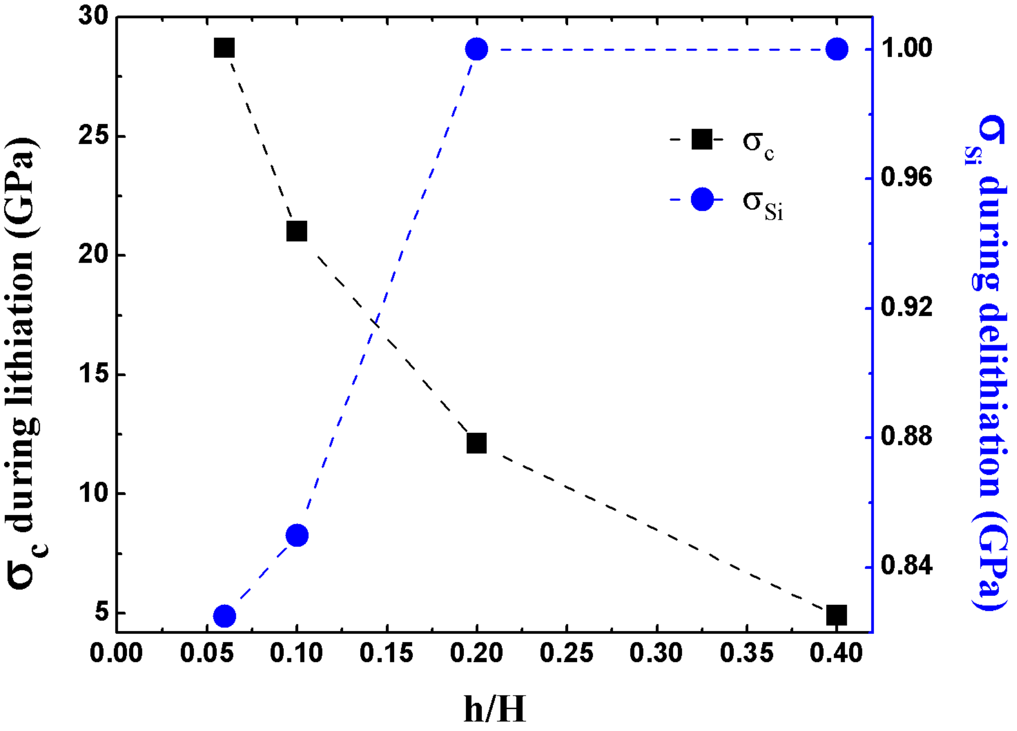

Supplementary Figure 22. In-plane stress analysis on 2DSi@C depending on the coating thickness. The first principal stress in the carbon coating layer (σ_c) during lithiation and that in the Si nanosheet (σ_{si}) during delithiation as a function of the coating thickness (h). Here H is the thickness of the Si nanosheet. Increasing the thickness of the coating layer reduces the tensile stress in the coating layer during lithiation but increases the tensile stress in the Si nanosheet during delithiation, which suggests the existence of an optimal thickness at which the tensile stresses in both the coating layer and the Si nanosheet are controlled in levels that avoid fracture.

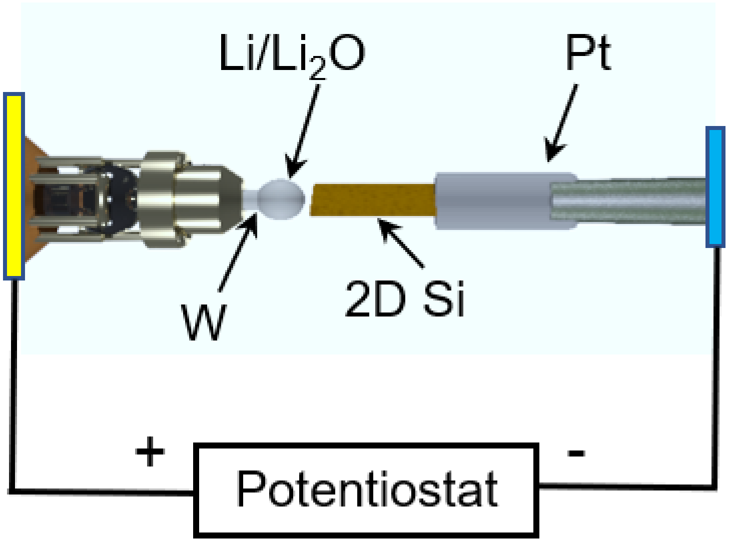

Supplementary Figure 23. Set up of nanobattery for *in situ* TEM observation.

Supplementary Notes

Supplementary Note 1. Synthesis of 2DSi via recyclable salt templating method

Salt templates provide a straightforward route for various structural modifications (hollow or 2D structures) on electroactive materials mainly including the metal oxides and carbon derivatives.[1-3] Particularly, sodium chloride (NaCl) serves as a sacrificing template owing to different water solubility between salt and target materials, while there has been no attempt to control its dimensions and improve the recycling efficiency at once. Furthermore, these products were not intended to meet the requirements for battery applications. By contrast, the method presented here aims at developing the battery-grade 2DSi anodes based on highly recyclable salt template of which size is readily tunable through its solubility control in rather scalable way.

Schematic illustration in **Supplementary Fig. 1a** depicts a large-scale synthetic process of 2DSi using cost-effective recyclable inorganic templates (less than 10 μm, **Supplementary Fig. 1b**) of 500 g per batch. Thermal decomposition of silane (SiH_4) gas at 550 °C produced the uniform Si-coated NaCl crystals (Si@NaCl, **Supplementary Fig. 1**c) without any deformation on templates. Subsequent washing process selectively dissolved the NaCl and the final 2DSi sheets insoluble in water were obtained by filtration (**Figure 1**a). The residual filtrate which contained dissolved salt templates recrystallized through adding a non-solvent and was recycled in this manner for the following 10 cycles with less than 1% loss as shown in **Supplementary Fig. 1**d. During this recrystallization and recycles, the size of commercial NaCl salt could be greatly reduced to micron scale, in which determined in-plane dimension of 2DSi materials. Non-solvent induced recrystallization process yields six facets of cubic NaCl with an average crystal size of 10 μm, and consequently the resulting 2D Si has a similar dimension which is quite suitable range (<20 μm)[4] for active materials in battery applications (**Supplementary Fig. 1**e).

Supplementary Note 2. Thickness-dependent electrochemical performances of 2DSi-based anodes

While maintaining a flat film-like morphology, 2DSi nanosheets with different thickness (50, 100, 150, and 380 nm) can be prepared by controlling the decomposition time of silane at a fixed temperature of 550 °C (**Supplementary Figs. 2, 3**a). Without fundamental information for 2DSi anodes, it was assumed that out-of-plane size of sheets solely affected its electrochemical behaviors, since in-plane size would be virtually homogenized during slurry formation. Despite nano-thick structure, 2DSi nanosheets have relatively low surface area of 2.42 m^2 g^{-1} for 380 nm 2D Si and 10.67 m^2 g^{-1} for 50 nm 2D Si, compared to other nanomaterials, which is ascribed to extensive in-plane size with negligible pores typically observed in CVD process (**Supplementary Fig. 3**b).[5] This leads to a high reversibility of ~90% and high reversible capacities over 2000 mAh g^{-1} at the initial galvanostatic test except the case of 380 nm-thick 2D Si, which have low ICE value of 46.3% resulting from the thicker frames above the fracture point of Si anodes (**Supplementary Fig. 3**c).

Our 2DSi nanosheet electrodes achieved the highest ICE value among previously reported 2DSi-based anodes as well as the improved capacity retention of ~95% after 200 cycles at 0.2 C-rate as shown in **Supplementary Fig. 3**d. As expected, the thinner 2DSi anodes showed the most promising electrochemical performances in terms of ICE, retentions, and rate capability (**Supplementary Fig. 3**e). These achievements are attributed to rational material design based on 2DSi sheets, through which the nano-thick structure enables fast Li-ion diffusion and micron-scale breadth with non-porous feature leads to low surface-to-volume ratio, suppressing the side reactions. In such a way, the 2DSi sheet anodes could go beyond a trade-off point between high ICE and rate capability/cycle stability. Li-ion kinetics through the nano-thick structure of 2DSi anodes strongly depend on its out-of-plane size (thickness), which can be validated by calculating the electrochemical active surface area of each electrode from the cyclic voltammogram based on Randles-Sevcik equation (**Supplementary Figs. 3**f, **4**). The slope values combined with other parameters showed that electrochemically activated surface area of 50 nm-thick 2DSi electrodes increased by 57 times with respect to geometrical surface, compared to 380 nm-thick 2DSi electrode. Given that 50 nm-thick 2DSi has higher surface area and relatively larger amount of electrolytes were decomposed, it is worth noticing that the formation of uniform SEI layers facilitates Li-ion diffusion. At least for 2DSi anodes, out-of-

plane size of sheets should be controlled as thick as 50 nm for further stuides of in situ TEM analysis and simulation works due to its high ICE, extended cycle life, and fast Li-ion kinetics.

Supplementary Note 3. Carbon coating thickness dependent rippling structure and in-plane stress analysis.

The bending energy scales $\Pi_B \sim E_c h^3 \kappa^2$, where E_c, h and κ are the Young's modulus, thickness, and curvature of the coating layer, respectively. The curvature of the coating layer scales $\kappa \sim A/\lambda^2$, where A is the amplitude and λ is the wavelength of the ripples[6]. Though the energy form of the substrate is quite complex and analytically unavailable (which prevents us from obtaining an analytical solution of A and λ in terms of other material properties), the bending energy of the coating layer suggests that a thick coating layer suppresses/disfavors the rippling (small κ and A) while a thin coating favors rippling of large amplitude (large κ and A). Thus, as the coating thickness increases to a certain point, rippling would not occur ($A \rightarrow 0$). In this case, the in-plane compressive energy of the carbon coating cannot be relaxed.

For in-plane stress analysis, we focused on either carbon coating layers or Si nanosheet depending on where the tensile stress is applied to. During lithiation, the coating layer is subjected to tension while the Si in compression. As tension has potential to cause facture, we focus our analysis on the coating layer. Assuming the lithiation induced that mismatched strain is ϵ_m, the mismatch generates a tensile stress in the coating layer that approximately scales $\sigma_c \sim \epsilon_m/h$ (**Supplementary Fig. 22**). For very thin coating, σ_c could well exceed the fracture strength of the coating, causing cracking in the coating.

During delithiation, the coating layer is subjected to compression while the Si nano sheet in tension, so we focus our analysis on the Si nano sheet. The tensile stress of the nanosheet in the post buckling state of the coating is very complex and analytically unviable. However, one can easily see that the in-plane stress of the Si nano-sheet scales by $\sigma_I^{Si} \sim E_{Si}(\epsilon_0 - A^2/\lambda^2)$, where ϵ_0 is the chemical strain due to the delithiation inside the Si nanosheet, and A^2/λ^2 is the strain accommodated by the rippling morphology. The difference between the two is the actual tensile strain of the fully delithiated state. As discussed in **Supplementary Fig. 21**, thicker coating layer corresponds to smaller rippling amplitude (smaller A/λ), which causes very large in-plane tensile stress inside the Si nanosheet.

Supplementary Table 1. Comparison of electrochemical performances of 2DSi-based anodes and other nano-engineered Si anodes.

Electrodes	ICE(%)	Current density ($A\ g^{-1}$)	Capacity ($mAh\ g^{-1}$) (after X cycles) Retention (%)	Loading levels ($mg\ cm^{-2}$)	References
SiMP@Graphene	93.2	1.5	1,400 (500)<br>85	0.8	30[a]
Pomegranate Si/C	82	0.7-1.0	1,160 (1,000)<br>97	0.2	19[a]
Hollow Si	77	1.5	1,420 (700)<br>57	0.1	17[a]
Si-C yolk-shell	60	3.0	1,500 (1,000)<br>74	1.0	36[a]
Si-embedded graphite/carbon hybride	92	0.25	500 (100)<br>96	6.5	18[a]
Si/C secondary particle	83.5	4.0	1,243 (150)<br>91	1.0	7[b]
Ultrathin Si nanosheet	79.4	3.0	865 (700)<br>92.3	2.12	14[a]
Carbon-coated Si nanosheets	47.7	0.4	1,575.5 (500)<br>92	1.5	8[b]
2DSi@C	92.3	0.4	1,914 (200)<br>94.9	0.5-1.1	This work
		2.0	1,145 (500)<br>62.6		

Note: [a]References in the manuscript and [b]References in the Supplementary Information. The displayed capacities are based on the active materials only.

Supplementary References

1. Liu, R. *et al.* Sodium Chloride Template Synthesis of Cubic Tin Dioxide Hollow Particles for Lithium Ion Battery Applications. *ACS Appl. Mater. Interfaces* **4**, 1537–1542 (2012).
2. Xiao, X. *et al*. Scalable Salt-Templated Synthesis of Two-Dimensional Transition Metal Oxides. *Nat. Commun.* **7**, 11296 (2016).
3. Shi, L. *et al*. Direct Synthesis of Few-Layer Graphene on NaCl Crystals. *Small* **11**, 6302–6308 (2015).
4. Huang, T. et al. Linking Particle Size to Improved Electrochemical Performance of SiO Anodes for Li-Ion Batteries. *RSC Adv.* **7**, 2273–2280 (2017).
5. Tiwari, J. N., Tiwari, R. N. & Kim, K. S. Zero-Dimensional, One-Dimensional, Two-Dimensional and Three-Dimensional Nanostructured Materials for Advanced Electrochemical Energy Devices. *Prog. Mater. Sci.* **57**, 724–803 (2012).
6. Efimenko, K. et al. Nested self-similar wrinkling patterns in skins. *Nat. Mater.* **4**, 293–297 (2005).
7. Jung, D. S., Hwang, T. H., Park, S. B. & Choi, J. W. Spary Drying Method for Large-Scale and High-Performance Silicon Negative Electrodes in Li-Ion Batteries. *Nano Lett.* **13**, 2092–2097 (2013).
8. Chen, S. *et al.* Scalable 2D Mesoporous Silicon Nanosheets for High-Performance Lithium-Ion Battery Anode. *Small* **14**, 1703361 (2018).
