# Supplementary material for: Mechanical mismatch-driven rippling in carbon-coated silicon sheets for stress-resilient battery anodes
Source: Nat Commun. 2018 Jul 26;9:2924. doi: 10.1038/s41467-018-05398-9 (PMC6062545; doi:10.1038/s41467-018-05398-9)
Supplement: Supplementary file 3 — Description of Additional Supplementary Files [file 41467_2018_5398_MOESM3_ESM.pdf]

Description of Additional Supplementary Files

File Name: Supplementary Movie 1
Description: First lithiation of 2D Si@C under bias of -3.0 V. The frame speed is 32X times of the real time.

File Name: Supplementary Movie 2
Description: First delithiation of 2D Si@C under bias of +3.0 V. The frame speed is 32X times of the real time.

File Name: Supplementary Movie 3
Description: Second lithiation of 2D Si@C under bias of -3.0 V. The frame speed is 16X times of the real time.

File Name: Supplementary Movie 4
Description: First lithiation of bare 2D Si under bias of -3.0 V. The frame speed is 64X times of the real time.

File Name: Supplementary Movie 5
Description: First delithiation of bare 2D Si under bias of +3.0 V. The frame speed is 64X times of the real time.
